# Association of metabolic syndrome with depression in US adults: A nationwide cross-sectional study using propensity score-based analysis

**DOI:** 10.3389/fpubh.2023.1081854

**Published:** 2023-02-01

**Authors:** Li Zhang, Quan Zhou, Li Hua Shao, Xue Qin Hu, Jun Wen, Jun Xia

**Affiliations:** ^1^Department of Neurology, The First People's Hospital of Changde, Changde, Hunan, China; ^2^Department of Science and Education, The First People's Hospital of Changde, Changde, Hunan, China; ^3^Department of Neurosurgery, The First People's Hospital of Changde, Changde, Hunan, China

**Keywords:** metabolic syndrome, depression, propensity score-based analysis, positive association, cross-sectional study

## Abstract

**Background:**

The association of metabolic syndrome (MetS) with depression has been previously reported; however, the results are ambiguous due to imbalanced confounding factors. Propensity score-based analysis is of great significance to minimize the impact of confounders in observational studies. Thus, the current study aimed to clarify the influence of MetS on depression incidence in the U.S. adult population by using propensity score (PS)-based analysis.

**Methods:**

Data from 11,956 adults aged 20–85 years from the National Health and Nutrition Examination Survey (NHANES) database between 2005 and 2018 were utilized. Using 1:1 PS matching (PSM), the present cross-sectional study included 4,194 participants with and without MetS. A multivariate logistic regression model and three PS-based methods were applied to assess the actual association between MetS and depression incidence. Stratified analyses and interactions were performed based on age, sex, race, and components of MetS.

**Results:**

After PSM, the risk of developing depression in patients with MetS increased by 40% in the PS-adjusted model (OR = 1.40, 95% confidence interval [CI]: 1.202–1.619, *P* < 0.001), and we could still observe a positive association in the fully adjusted model (OR = 1.37, 95% CI: 1.172–1.596, *P* < 0.001). Regarding the count of MetS components, having four and five conditions significantly elevated the risk of depression both in the PS-adjusted model (OR = 1.78, 95% CI: 1.341–2.016, *P* < 0.001 vs. OR = 2.11, 95% CI: 1.626–2.699, *P* < 0.001) and in the fully adjusted model (OR = 1.56, 95 CI%: 1.264–1.933, *P* < 0.001 vs. OR = 1.90, 95% CI: 1.458–2.486, *P* < 0.001). In addition, an elevation in MetS component count was associated with a significant linear elevation in the mean score of PHQ-9 (F =2.8356, *P* < 0.001). In the sensitivity analysis, similar conclusions were reached for both the original and weighted cohorts. Further interaction analysis revealed a clear gender-based difference in the association between MetS and depression incidence.

**Conclusion:**

MetS exhibited the greatest influence on depression incidence in US adults, supporting the necessity of early detection and treatment of depressive symptoms in patients with MetS (or its components), particularly in female cases.

## Introduction

Major depression, characterized by limited psychosocial function and a reduction in the quality of life, is expected to become the third largest cause of the overall disease burden worldwide (1). In the past 30 years, the number of global depression cases increased by 49.86% (2), causing a huge economic burden. Previous studies indicated several risk factors associated with depression, including old age, female gender, low education level, cognitive impairment, central obesity, physiological abnormalities, and a chronic medical history (3–5). A deeper excavating of the impact of risk factors on depression is of advantage to effective prophylaxis and cure. Aside from these traditional risk factors, the influence of metabolic syndrome (MetS) on the development of depression should be studied.

MetS has been proposed as a risk factor for cardiovascular disease (CVD). Patients diagnosed with MetS are at a greater risk of CVD (2–3 times) and type 2 diabetes (five times) (6). Insulin resistance (IR) is thought to be the core mechanism of MetS. Patients with depression could also show IR and glucose intolerance (7). Meanwhile, arterial stiffness (AS), a key mediator of CVD, was confirmed to be associated with middle-aged depression (5). Patients with mental illness have a higher prevalence of MetS, ranging from 29.4 to 67.9% (8). In Brazil's general population, the risk of MetS in patients with psychiatric disorders was 1.58 times higher (8, 9). All these findings suggested that there should be a link between MetS and depression.

Recently, several scholars have paid attention to the depression–MetS relationship, but the results obtained were still ambiguous. A few studies supported the independent correlation of MetS with elevated depression risk, even after adjusting for related factors (10–12), whereas other studies have not found any positive association between mental distress and MetS (13, 14). Scholars have frequently utilized traditional regression models to control for confounding. However, such methods may cause bias due to unmeasured or residual confounders, while including all available variables may cause the model to overfit, preventing the effective identification of the association between exposures of interest and outcome (15). The adjustment method based on the propensity score (PS) is of great significance to limit confounding in observational studies. It was pointed out that adjusting PS is important to eliminate biases caused by all observational covariates (16, 17). Scholars have attempted to introduce weighting, regression adjustment, and matching as PS-based adjustment methods (17).

The present study aimed at evaluating the actual association between MetS and depression incidence using PS-based analysis in US adults aged 20–85 years, utilizing data from the National Health and Nutrition Examination Survey (NHANES) over the period 2005–2018.

## Methods

### Study design and data source

The data of this study were obtained from NHANES, as previously described (18). The NHANES survey contained two parts; a family interview covering demographics, socioeconomic, nutritional, and health concerns; and a routine physical examination completed at the Mobile Examination Center (MEC) involving medical, dental, physiological measures, and laboratory testing. More details on NHANES can be obtained from the database.

**Figure 1:**
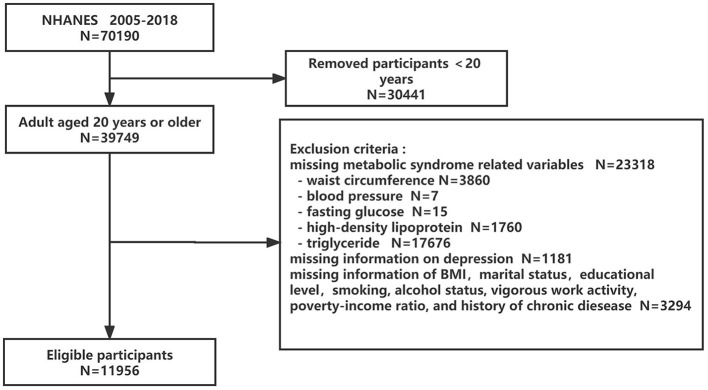
Flowchart of the eligible participant selection process. NHANES, National Health and Nutrition Examination Surveys.

Seven cycles of continuous NHANES data (2005–2018) were pooled in this cross-sectional study to produce sizable samples for analysis. Of the 70,190 subjects, we first eliminated individuals younger than 20 years (*n* = 30,441), followed by those who had missing data for MetS components (*n* = 23,318), depression questionnaire (*n* = 1,181), and other confounding factors (*n* = 3,294). Finally, this study contained 11,956 eligible subjects. The flowchart for choosing eligible subjects is displayed in Figure 1. The NCHS Research Ethics Review Committee gave its approval for all data collection, and all participants gave their written informed permission.

### Assessment of depressive symptoms

We utilized the Patient Health Questionnaire-9 (PHQ-9) scores to evaluate depressive symptoms (19). A total score of ≥ 10 was used as the cutoff to define depression according to a previous study (19).

### Assessment of metabolic factors

We evaluated waist circumference with the assistance of trained NHANES staff through procedures designed for this target. Blood pressure was measured by an automatic sphygmomanometer at rest, and the mean value of three right-arm readings was recorded. Laboratory data for fasting glucose, HDL-C, and triglyceride levels were determined from fasting plasma samples using routine methods. More information on sample collection and processing instructions can be obtained from the NHANES Laboratory Procedures Manual.

### Definition of metabolic syndrome

According to the revised National Cholesterol Education Program-Adult Treatment Panel III (NCEP-ATP III) criteria (20), MetS was defined as having at least three of the following conditions: (1) abdominal obesity, defined as a waist circumference of at least 102 cm for men and at least 88 cm for women; (2) hypertension, defined as an SBP of ≥130 mmHg or a DBP of ≥85 mmHg, or pharmacological therapy; (3) hypertriglyceridemia, defined as a triglyceride level of ≥150 mg/dL or fibrates being used; (4) low HDL-C level, defined as an HDL-C of <40 mg/dL in men and <50 mg/dL in women or having recently used lipid-lowering drugs; (5) hyperglycemia, defined as a fasting plasma glucose level of ≥100 mg/dL or currently using insulin or oral hypoglycemic drugs (20).

### Collection of confounding factors

As potential confounders, the sociodemographic factors, lifestyle factors, and the health examination of subjects were collected, which including age, sex, race, marital status, education level, poverty-to-income ratio (PIR), smoking status, alcohol status, vigorous work activity, and history of chronic diseases [congestive heart failure (CHF), coronary heart disease (CHD), angina, heart attack, hypertension, diabetes mellitus, hyperlipidemia, and stroke]. PIR was stratified as ≤1.3, 1.3–1.85, and >1.85, based on data from the original survey. Smoking status was categorized as current, former, and never based on participants' answers to the following questions: “Have you smoked more than 100 cigarettes in your lifetime?” and “Do you smoke now?” Alcohol status was regarded as positive if participants consumed ≥12 alcoholic drinks per year. Vigorous work activity was defined in terms of responses to participation in the vigorous-intensity activity. A history of chronic diseases was based on self-reports of physician diagnoses.

### Statistical analysis

Continuous variables were presented as the mean±standard deviation (SD), and comparison between groups was made using the two-sample *t*-test; the expression of categorical variables was undertaken as percentages, and the statistical differences between groups were measured using the Rao-Scott chi-square test.

PS analysis matched all confounding variables listed in Table 1 between MetS and non-MetS groups, and a single group, involving subjects with similar covariates, was formed. A non-parsimonious multivariable logistic regression model was utilized to estimate PS, in which MetS and 14 confounding variables were regarded as independent variables and covariates, respectively. A 1:1 greedy nearest neighbor matching without replacement (greedy matching algorithm) was performed in this study, and the caliper width was set to 0.01. Standardized difference (SD) was calculated as the evaluation index of the covariate balance in these matched participants. For a given covariate, a SD <10.0% represents a relatively little imbalance (21, 22). Detailed information on PSM is provided in Supplementary Table 1 and Supplementary Figure 2. In addition, an assessment of differences in the PHQ-9 mean score was undertaken using Fisher's exact method by counting MetS components.

**Table 1:** Baseline characteristics of MetS and non-MetS participants before and after propensity score matching.

**Variables**	**Before Matching**				**After Matching**			
	**MetS**	**Non-MetS**	**SD** **(100%)**	***P*** **-value**	**MetS**	**Non-MetS**	**SD** **(100%)**	***P*** **-value**
**Participants**	4,719	7,237			4,194	4,194		
**Age, years**	55.62 ± 15.92	44.94 ± 17.48	63.9	< 0.001	53.82 ± 15.70	52.94 ± 17.08	5.3	0.014
**Sex**			1.6	0.383			3.4	0.121
Men, *n* (%)	2,305 (48.85)	3,594 (49.66)			2,072 (49.40)	2,143 (51.10)		
Women, *n* (%)	2,414 (51.15)	3,643 (50.34)			2,122 (50.60)	2,051 (48.90)		
**Race**			17.1	< 0.001			9.3	0.001
Mexican American, *n* (%)	763 (16.17)	1,095 (15.13)			678 (16.17)	748 (17.84)		
Non-Hispanic black, *n* (%)	901(19.09)	1,432 (19.79)			822 (19.60)	761 (18.15)		
Non-Hispanic white, *n* (%)	2,310 (48.95)	3,257 (45.00)			2,027 (48.33)	1,918 (45.73)		
Other Hispanic, *n* (%)	449 (9.51)	665 (9.19)			390 (9.30)	418 (9.97)		
Other race or multi-racial, *n* (%)	296 (6.27)	788 (10.89)			277 (6.61)	349 (8.32)		
**Marital status**			36.3	< 0.001			4.8	0.184
Married/living with a partner, *n* (%)	2,952 (62.56)	4,357 (60.20)			2,642 (63.00)	2,688 (64.09)		
Widowed, *n* (%)	517 (10.96)	376 (5.20)			399 (9.51)	354 (8.44)		
Divorced/separated, *n* (%)	751 (15.91)	922 (12.74)			669 (15.95)	637 (15.19)		
Never married, *n* (%)	499 (10.57)	1,582 (21.86)			484 (11.54)	515 (12.28)		
**Education level**			28.1	< 0.001			11.3	< 0.001
Less than high school, *n* (%)	1,347 (28.54)	1,543 (21.32)			1,117 (26.63)	1,208 (28.80)		
High school graduate/GED or equivalent, *n* (%)	1,170 (24.79)	1,540 (21.28)			1,026 (24.46)	979 (23.31)		
Some college or AA degree, *n* (%)	1,378 (29.20)	2,107 (29.11)			1,267 (30.21)	1,096 (26.13)		
College graduate or above, *n* (%)	824 (17.46)	2,047 (28.29)			784 (18.69)	911 (21.72)		
**Poverty-income ratio**			11.7	< 0.001			2.0	0.651
≤ 1.3	1,602 (33.95)	2,122 (29.32)			1,372 (32.71)	1,405 (33.50)		
>1.3, ≤ 1.85	645 (13.67)	908 (12.55)			572 (13.64)	581 (13.85)		
>1.85	2472 (52.38)	4207 (58.13)			2250 (53.65)	2208 (52.65)		
**Smoking status**			20.7	< 0.001			0.9	0.919
Current smoker, *n* (%)	953 (20.19)	1,535 (21.21)			889 (21.20)	884 (21.08)		
Former smoker, *n* (%)	1,443 (30.58)	1,571 (21.71)			1,191 (28.40)	1,208 (28.80)		
Never smokers, *n* (%)	2,323 (49.23)	4,131 (57.08)			2,114 (50.41)	2,102 (50.12)		
**Alcohol status**, *n* (%)	3,260 (69.08)	5,374 (74.26)	11.5	< 0.001	2,958 (70.53)	2,991 (71.32)	1.7	0.428
**Vigorous work activity**, *n* (%)	900 (19.07)	1,674 (23.13)	10.0	< 0.001	852 (20.32)	938 (22.37)	5.0	0.022
**History of chronic diseases**								
CHF, *n* (%)	251 (5.32)	100 (1.38)	22.0	< 0.001	150 (3.58)	98 (2.34)	7.3	< 0.001
CHD, *n* (%)	298 (6.31)	166 (2.29)	19.9	< 0.001	208 (4.96)	162 (3.86)	5.3	0.014
Angina, *n* (%)	197 (4.17)	97 (1.34)	17.4	< 0.001	124 (2.96)	96 (2.29)	4.2	0.056
Heart attack, *n* (%)	311 (6.59)	157 (2.17)	21.7	< 0.001	201 (4.79)	154 (3.67)	5.6	0.011
Stroke, *n* (%)	257 (5.45)	162 (2.24)	16.7	< 0.001	183 (4.36)	151 (3.60)	3.9	0.074

AA, Associate of Arts; MetS, metabolic syndrome; GED, General Equivalent Diploma; CHF, congestive heart failure; CHD, coronary heart disease.

In our study, a robust estimation method was applied to control confounding variables and to evaluate the actual association between MetS and depression incidence. Specifically, the multiple logistic regression model and three PS-based models were used, including PS matching, PS adjustment, and inverse probability of treatment-weighted (IPTW) models were employed. First, the multiple logistic regression models were designed by adjusting for covariates in the PS-matched cohort. Second, PS adjustment was defined as a multivariate-adjusted regression model with adjustment for PS in the PS-matched cohort (17, 23). Third, for sensitivity analyses, an estimation of PS was undertaken for the calculation of IPTW. For instance, 1/PS was regarded as the weight of MetS, while 1/(1–PS) was attributable to the weight of non-MetS. The creation of a weighted cohort was undertaken *via* the IPTW model (24, 25). Using two relationship inference models (both in the original and weighted cohorts), sensitivity analysis was conducted. In addition, because PSM could only control the influence of measured confounders, if there are still unmeasured confounding factors, this will bring invisible bias. The E-value was calculated to assess the possibility of unmeasured confounders affecting the observed association between MetS and depression (26).

To further determine the robustness of our results in the diverse subgroups, stratified analysis based on age, sex, race, and components of MetS was also performed using stratified multivariate regression models. Each stratification was adjusted for PS in the PS-matched cohort. Exploration of modifications and interactions of subgroups was carried out using likelihood ratio tests. The STROBE statement was utilized to report the findings (27). R programming (version 4.1.3) and Empower Stats 4.1 software were applied for statistical analysis, and P < 0.05 was considered for defining statistical differences.

## Results

### Baseline characteristics

In this study, we enrolled 11,956 eligible subjects, whose mean age was 49.15 [17.67] years. Among them, 1,003 (8.4%) suffered from depression, with 369 (6.3%) and 634 (10.5%) being men and women, respectively. Before PSM, between the MetS group versus the non-MetS group, we identified significant differences in several confounding variables (Table 1). Patients with MetS appeared to be older, widowed, or divorced, with a lower PIR and education level, had a higher prevalence of cardiac–cerebral vascular diseases, and were more likely to be former smokers and drinkers. In general, 4,194 patients with MetS were successfully matched with non-MetS subjects by using a 1:1 PSM. Except for educational level, almost computation of standardized differences (SDs) indicated a rate of <10% for almost all covariates, exhibiting a well-matched after PSM.

### MetS and its components exhibited a correlation with depression

We utilized multiple logistic regression models to clarify whether MetS is correlated with depression incidence after PSM. In the non-adjusted model, patients with MetS presented with a higher risk of depression (Model 1: OR: 1.41, 95 CI%: 1.211–1.630, *P* < 0.001; Table 2). After adjusting for all confounding factors, the results of Model 2 (OR:1.37, 95 CI%: 1.172–1.596, *P* < *0.001*) were similar to those of Model 1. Even in the PS-adjusted model, the incidence of depression was still higher in patients with MetS (Model 3: OR: 1.40, 95 CI%: 1.202–1.619, *P* < *0.001*). In addition, we also found a greater risk of depression among those cases whose count of MetS components was higher. Compared with those who had less than three MetS components, participants who had four or five components of MetS were 1.56 and 1.90 times, respectively, more likely to develop depression after full adjustment (Model 2: OR: 1.56, 95% CI: 1.264–1.933, *P*<* 0.001 * vs. OR: 1.90, 95% CI: 1.458–2.486, *P* < *0.001*). The association still existed after adjusting for PS (Model 3: OR: 1.78, 95 CI%: 1.341–2.016, *P*<*0.001* vs. OR: 2.11, 95% CI: 1.626–2.699, *P*<*0.001*). To ensure the robustness of our results, we also handled the count of MetS components as a continuous variable and observed the same trend (*P* for the trend < *0.001*).

**Table 2:** Adjusted odds ratios for the prevalence of depression according to the presence of metabolic syndrome and its components in the PS-matched cohort.

**Variable**	**Model 1**		**Model 2**		**Model 3**	
	**OR (95%CI)**	***P*** **-value**	**OR (95%CI)**	***P*** **-value**	**OR (95%CI)**	***P*** **-value**
**MetS**						
NO	Ref		Ref		Ref	
YES	1.41 (1.211-1.630)	< 0.001	1.37 (1.172-1.596)	< 0.001	1.40 (1.202-1.619)	< 0.001
**Components of MetS**						
< 3	Ref		Ref		Ref	
3	1.12 (0.933-1.338)	0.2284	1.14 (0.945-1.372)	0.1722	1.12 (0.933-1.339)	0.2266
4	1.66 (1.354- 2.035)	< 0.001	1.56 (1.264-1.933)	< 0.001	1.78 (1.341-2.016)	< 0.001
5	2.16 (1.676- 2.776)	< 0.001	1.90 (1.458-2.486)	< 0.001	2.11 (1.626-2.699)	< 0.001
*P*-value for the trend		< 0.001		< 0.001		< 0.001

OR, odds ratio; CI, confidence interval. Model 1 no adjustement for other covariates. Model 2 adjusted for age, sex, race, marital status, educational level, PIR, smoking status, alcohol status, vigorous work activity, congestive heart failure, coronary heart disease, angina, heart attack, and stroke. Model 3 adjusted for propensity score.

**Figure 2:**
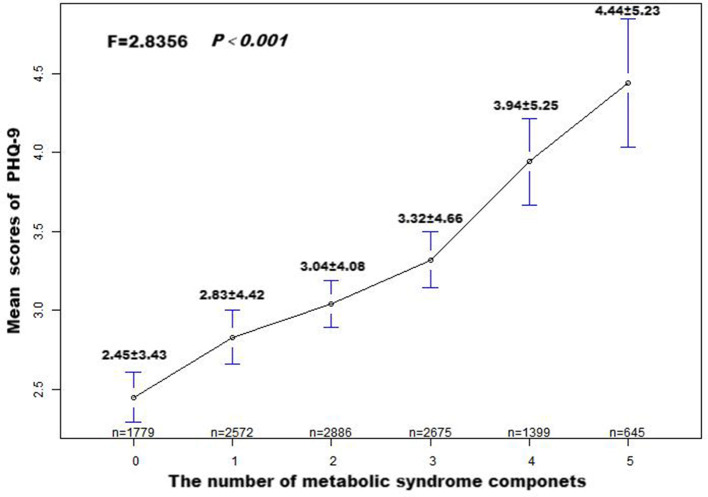
Patient Health Questionnaire-9 (PHQ-9) means score and standard error, according to the number of metabolic syndrome components. *P*-values were obtained by Fisher's exact test.

The patients' mean PHQ-9 score, based on MetS components count, is presented in Figure 2. It was noted that an elevation in MetS components count was associated with a significant linear elevation in the mean score of PHQ-9 (F = 2.8356, *P* < *0.001*).

### Sensitivity analysis

This study applied sensitivity analysis to further confirm the association between MetS and the incidence of depression in the two mentioned cohorts (Table 3). We utilized the estimated PS to form a weighted cohort *via* developing an IPTW model. In addition, we attempted to use the non-adjusted, partially adjusted, and fully adjusted models in both cohorts. A greater risk of developing depression was strongly related to patients with MetS in the cohorts. After adjusting all covariates, a greater risk of MetS was noted in those cases with MetS (Model 3: OR: 1.44, 95% CI: 1.247–1.659, *P* < *0.001*) in the original cohort, and the results (Model 3: OR: 1.43, 95% CI: 1.241–1.642, *P* < *0.001*) remained marked in the weighted cohort. MetS components count and the increased incidence of depression exhibited an independent relationship in the two cohorts. After adjusting all covariates, patients in the two cohorts with four (Model 3: OR: 1.69, 95% CI:1.390–2.058, *P* < *0.001* vs. OR: 1.70, 95% CI: 1.440–2.010, *P* < *0.001*) or five (Model 3: OR: 1.94, 95% CI:1.507–2.496, *P* < *0.001* vs. OR: 2.07, 95% CI: 1.697–2.535, *P* < *0.001*) components of MetS had a significantly elevated risk of depression than those without.

**Table 3:** Association of MetS and its components with depression in the original and the weighted cohorts.

**Variable (A)**	**Model 1**		**Model 2**		**Model 3**	
	**OR (95%CI)**	***P*** **-value**	**OR (95%CI)**	***P*** **-value**	**OR (95%CI)**	***P*** **-value**
**Non-MetS**	Ref		Ref		Ref	
**MetS**	1.69 (1.482-1.920)	< 0.001	1.49 (1.291-1.713)	0.0010	1.44 (1.247-1.659)	< 0.001
**Components of MetS**						
< 3	Ref		Ref		Ref	
3	1.32 (1.124-1.556)	< 0.001	1.22 (1.024-1.447)	0.0255	1.19 (1.004-1.420)	0.0452
4	2.04 (1.700–2.443)	< 0.001	1.75 (1.444-2.131)	< 0.001	1.69 (1.390-2.058)	< 0.001
5	2.53 (2.007- 3.194)	< 0.001	2.07 (1.617-2.660)	< 0.001	1.94 (1.507-2.496)	< 0.001
*P*-value for the trend		< 0.001		< 0.001		< 0.001
**Variable (B)**	**Model 1**		**Model 2**		**Model 3**	
	**OR (95%CI)**	***P*** **-value**	**OR (95%CI)**	***P*** **-value**	**OR (95%CI)**	***P*** **-value**
**Non-MetS**	Ref		Ref		Ref	
**MetS**	1.64 (1.436-1.867)	< 0.001	1.46 (1.267-1.674)	< 0.001	1.43 (1.241-1.642)	< 0.001
**Components of MetS**						
< 3	Ref		Ref		Ref	
3	1.29 (1.115-1.490)	< 0.001	1.20 (1.026-1.392)	0.0217	1.19 (1.017-1.381)	0.0291
4	2.00 (1.709- 2.334)	< 0.001	1.74 (1.477-2.059)	< 0.001	1.70 (1.440-2.010)	< 0.001
5	2.53 (2.104- 3.045)	< 0.001	2.17 (1.779-2.648)	< 0.001	2.07 (1.697-2.535)	< 0.001
*P*-value for the trend		< 0.001		< 0.001		< 0.001

A: In the original cohort; B: in the weighted cohort. OR, odds ratio; CI, confidence interval. Model 1 no adjustement for other covariates. Model 2 adjusted for age, sex, race, marital status, educational level, PIR, smoking status, alcohol consumption status, and vigorous work activity. Model 3 adjusted for age, sex, race, marital status, educational level, PIR, smoking status, alcohol consumption status, vigorous work activity, congestive heart failure, coronary heart disease, angina, heart attack, and stroke.

Moreover, the sensitivity of unmeasured confounders was estimated *via* the calculation of the E-value. The E-value was 2.15 (lower confidence limit, 1.68), indicating that there is less likely to be an unmeasured confounding factor that can affect the current association between MetS and depression incidence.

### Subgroup analysis

**Figure 3:**
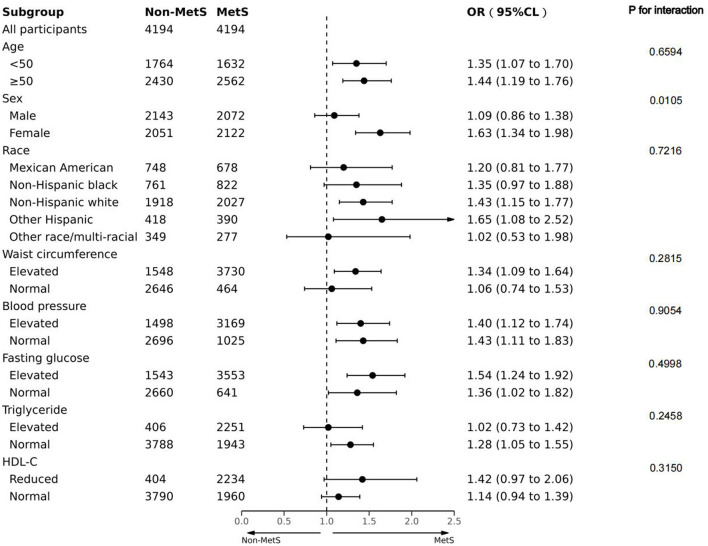
Forest plot of the association between MetS and depression in terms of age, sex, race, and components associated with MetS in the PS-matched cohort. Adjusted for PS.

We attempted to carry out a stratified analysis to assess the robustness of our findings in the diverse subgroups after PSM. Figure 3 indicates that stratified analysis based on the age, sex, race, and components of MetS yielded consistent outcomes. The interaction analysis revealed a clear gender-based difference in the relationship between MetS and depression incidence. After adjusting for possible confounders, the risk of depression in female participants with MetS was 1.63 times higher than those without (OR:1.63, 95 CI%: 1.34–1.98). However, the association did not reach a statistical difference between male participants with MetS and depression (*P* for the interaction < 0.05). No noticeable interaction was identified for age, race, or components of MetS.

## Discussion

Depression is one of the most prevalent mental illnesses affecting adults, seriously impacting public health in the USA (28). To date, the concept of MetS has been assessed, and was related to a greater likelihood of CVD and all-cause mortality (29). Previously, few studies have attempted to determine the association between MetS and the prevalence of depression. In this PSM cohort study, we observed a positive association between MetS and depression incidence in US adults: Patients with MetS had a 40% elevated risk of developing depression after adjusting for PS, and the association still existed after adjusting for all confounders. In addition, an elevated MetS components count was positively associated with a greater risk of developing depression after PSM. In the sensitivity analysis, similar conclusions were reached for both the original and weighted cohorts. Even after adjusting for PS, subgroup analysis stratified by the chosen variables yielded consistent findings. Further interaction analysis revealed a clear gender-based difference in the relationship between MetS and depression risk.

As a comorbid factor of multiple diseases, depression has been associated with all-cause mortality (30) and adverse health outcomes (31). Scholars have pointed out a link between depression and MetS. In a recent meta-analysis, a bi-directional association between the two was detected in prospective cohort studies (32), consistent with two other cross-sectional studies, which showed a link between depression and MetS in Korean adults (12) and the rural Chinese population (3). In another longitudinal cohort study, the utilization of antidepressants and elevated depressive symptoms exhibited a link with short-term metabolic dysregulation (33). In the current study, the positive association of MetS with depression occurrence was also observed. The interaction between MetS and depression may be regulated by multiple mechanisms. First, the pathophysiology of depression and Mets shared similar biological processes, involving central obesity (34), insulin resistance (35), and chronic inflammation (36); thus, the occurrence and development of MetS may increase the risk of depression. Second, according to the vascular depression hypothesis, vascular damage in the brain may be a predisposing factor to depression in the elderly population (37). Third, the common unhealthy lifestyle related to depression and MetS, such as poor diet and sleep, smoking and alcohol use, as well as physical inactivity, may contribute to the promotion and development of each (38, 39). Fourth, antidepressants may have direct impacts on MetS components, for example, the use of tricyclic antidepressants (TCA) is related to abdominal obesity; conversely, a negative self-perception due to abdominal obesity may increase the risk of depression (40). In short, the mechanisms underlying this interrelationship are complex and unclear, and more research is needed, which will be essential for the prevention and treatment of both conditions.

The current study demonstrated that MetS and depression incidence exhibited a positive relationship. In this study, after PSM, the odds ratio for depression in patients with MetS was 1.40 (95% CI = 1.202–1.619), within the range of 1.23 to 1.52, which was provided as the odds ratio of patients with MetS for developing depression in a systematic review (33), confirming the importance of early detection and treatment of depression in patients with MetS. In terms of MetS components count, we observed a positive relationship of elevated OR for depression with MetS components count. Consistently, a study from the Korean NHANE (2007–2013) showed that MetS components count and the increased risk of depression exhibited a relationship (10). In addition, previous studies have also shown that the greater the MetS components count, the higher the mean PHQ-9 score (12, 35) or the severity of depression (12). The findings indicated that active treatment of depression should not only be aimed at those diagnosed with MetS but also those with a higher number of MetS components.

In addition, a sex difference in the association between MetS and depression occurrence was detected in the current study. The finding that the association was more remarkable in female patients with MetS agreed with previous studies (41–43). Physiological hormone differences (44), distinct lifestyle habits (45), and the use of a self-reported symptom scale for depression may partly explain the sex difference in this association. Men were more likely to under-report the severity of their depression, resulting in a classification bias.

Although a growing body of evidence has well-established the cross-sectional relationship between psychopathology and metabolic dysregulation, our study was the first conducted to explore the association between MetS and depression incidence by using the PS-based method. In addition, we attempted to conduct a sensitivity analysis to prove that our findings were robust. Moreover, our results may be more convincing due to the national representation and large sample size of NHANES. Lastly, age, sex, race, and components of MetS were selected for stratified analysis to analyze in more detail the effects of different populations diagnosed with MetS on depression incidence. However, the shortcomings of the present study should be acknowledged. First, the validity of the results might be influenced because no structured diagnostic scale was utilized to identify depression. Second, no longitudinal causal relationship could be determined between MetS and depression, attributable to the cross-sectional design. Third, some information (smoking status, physical activity, and alcohol consumption) was based on the participants' self-reports, highlighting the inevitability of bias risk.

## Conclusion

In summary, MetS and depression incidence exhibited a positive relationship in a large, nationally representative study. The results highlighted the ongoing necessity for the early screening and management of depression in patients with MetS (or its components), particularly in female cases.

## Data availability statement

The datasets presented in this study can be found in online repositories. The names of the repository/repositories and accession number(s) can be found at: http://www.cdc.gov/nhanes.

## Ethics statement

The studies involving human participants were reviewed and approved by the NCHS Research Ethics Review Committee. The patients/participants provided their written informed consent to participate in this study.

## Author contributions

LZ and JX contributed to the drafting of the manuscript, the analysis, interpretation of the data, read, and approved the final manuscript. LZ contributed to the conception, critical revision of the manuscript, analysis, interpretation of data, and approved the final version of the submitted manuscript. All authors contributed to the article and approved the submitted version.
