# Supplementary material for: Association of metabolic syndrome with depression in US adults: A nationwide cross-sectional study using propensity score-based analysis
Source: Front Public Health. 2023 Feb 1;11:1081854. doi: 10.3389/fpubh.2023.1081854 (PMC9929360; doi:10.3389/fpubh.2023.1081854)
Supplement: Supplementary file 1 [file Table_1.DOCX]

Supplemental table 1: Propensity score parameter list

| The variables used in calculating the propensity score | Age, sex, race, marital status, education level, poverty-income ratio, smoking status, alcohol status, vigorous work activity, history of [chronic disease](javascript:;)s (CHF,CHD, angina, heart attack, and stroke) |
| --- | --- |
| Propensity score algorithm   1. Statistical   Matching method  Distance metric  Matching ratio  Use of replacement  Matching sample size | Logistic regression model  0.7002  Greedy matching within specified caliper distances  0.01  1:1  Without replacement  No Y=1: 4194 cases Total:8388 cases  No Y=0: 4194 cases |
