# Supplementary figures and images for: Association of metabolic syndrome with depression in US adults: A nationwide cross-sectional study using propensity score-based analysis
Source: Front Public Health. 2023 Feb 1;11:1081854. doi: 10.3389/fpubh.2023.1081854 (PMC9929360; doi:10.3389/fpubh.2023.1081854)

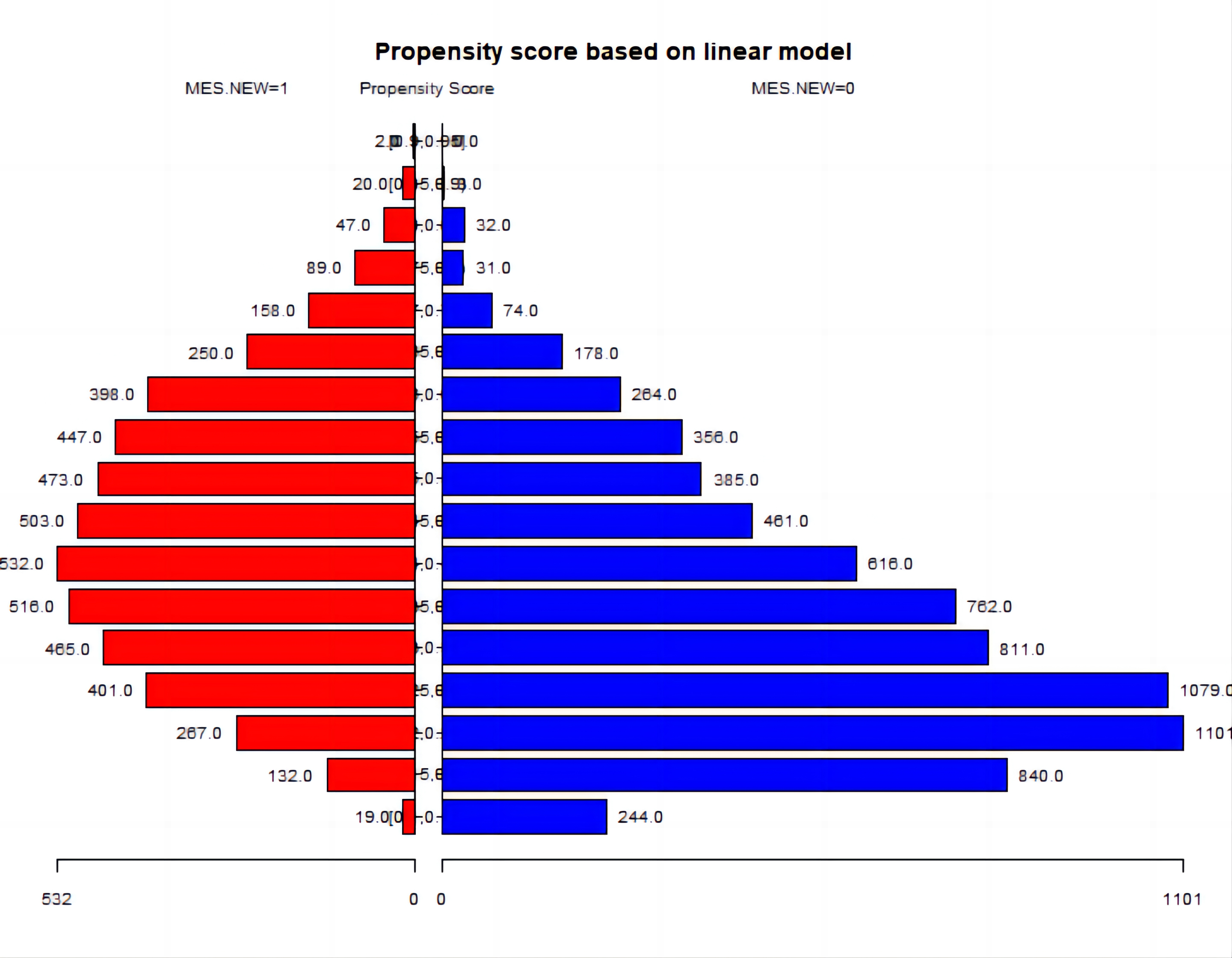

Supplement: Supplementary file 2 [file Image_1.jpg]
